# Perturbing the O–H^…^O Hydrogen Bond in 1-oxo-3-hydroxy-2-propene

**DOI:** 10.3390/molecules26113086

**Published:** 2021-05-21

**Authors:** Ibon Alkorta, José Elguero, Janet E. Del Bene

**Affiliations:** 1 Instituto de Química Médica, CSIC, Juan de la Cierva, 3, E-28006 Madrid, Spain; iqmbe17@iqm.csic.es; 2 Department of Chemistry, Youngstown State University, Youngstown, OH 44555, USA

**Keywords:** intramolecular hydrogen bonds, structures and binding energies, charge-transfer interactions, spin–spin coupling constants

## Abstract

Ab initio MP2/aug’-cc-pVTZ calculations have been carried out to identify and characterize equilibrium structures and transition structures on the 1-oxo-3-hydroxy-2-propene: Lewis acid potential energy surfaces, with the acids LiH, LiF, BeH_2_, and BeF_2_. Two equilibrium structures, one with the acid interacting with the C=O group and the other with the interaction occurring at the O–H group, exist on all surfaces. These structures are separated by transition structures that present the barriers to the interconversion of the two equilibrium structures. The structures with the acid interacting at the C=O group have the greater binding energies. Since the barriers to convert the structures with interaction occurring at the O–H group are small, only the isomers with interaction occurring at the C=O group could be experimentally observed, even at low temperatures. Charge-transfer energies were computed for equilibrium structures, and EOM-CCSD spin–spin coupling constants ^2h^J(O–O), ^1h^J(H–O), and ^1^J(O–H) were computed for equilibrium and transition structures. These coupling constants exhibit a second-order dependence on the corresponding distances, with very high correlation coefficients.

## 1. Introduction

1-oxo-3-hydroxy-2-propene, the enol of malonaldehyde in the conformation that presents an intramolecular hydrogen bond [1], has played an important role in the fields of hydrogen bonding, proton transfer [2,3,4,5,6,7,8], resonance assisted hydrogen bonds [6,9,10,11,12], quasi-aromaticity [13], and, in general, in non-covalent interactions. The importance of this molecule is due to the fact that it is the simplest of all 1,3-dicarbonyl compounds that were the first systems in which intramolecular hydrogen bonds had been studied [14,15,16,17,18,19].

Various aspects of 1-oxo-3-hydroxy-2-propene have been investigated, including substituent effects [20,21], solvent effects in which water molecules interact with this molecule [22], excited state properties [23], and the important problem of proton tunneling [23,24,25,26,27]. Both vibrational [28] and rotational [29,30,31] spectroscopic studies have been carried out on this molecule. In addition, interactions of 1-oxo-3-hydroxy-2-propene with Lewis acids such as methanol [32] and Li^+^, Na^+^ and FH [33], and BeX_2_ [34] have been reported.

A useful property for obtaining structural information about complexes linked by non-covalent interactions and, in particular by hydrogen bonds, is spin–spin coupling constants (SSCC). SSCC are related to the electronic structure of molecules and complexes through geometry, bond order, polarization, and electron densities. As Cremer and Gräfenstein wrote [35], “The analysis of NMR spin–spin coupling leads to a unique insight into the electronic structure of closed-shell molecules”. This was known for molecules from the beginning of the use of NMR spectroscopy [36,37,38] but was extended to complexes by Limbach [39,40] and Del Bene [41,42]. Through relationships between SSCC and geometry, the problem of the localization of the hydrogen-bonded proton could be solved [43].

In the present paper, we report the results of an investigation of 1-oxo-3-hydroxy-2-propene in a series of binary complexes with the acids LiH, LiF, BeH_2_, and BeF_2_. These complexes contain intramolecular O–H···O hydrogen bonds and lithium (alkali) and beryllium (alkaline earth) intermolecular bonds [44]. Specifically, we have determined the structures and binding energies of these complexes; the proton transfer barriers; the complex stabilization by charge-transfer interactions; and the spin–spin coupling constants ^2h^J(O–O), ^1h^J(H–O), and ^1^J(O–H) across the O–H**^…^**O hydrogen bond. It is the purpose of this paper to present and discuss the results of this study.

## 2. Results and Discussion

**Scheme 1:**
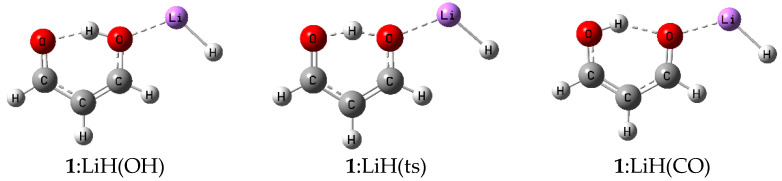
Some representative complexes.

In order to simplify the discussion of the equilibrium and the transition structures on the 1-oxo-3-hydroxy-2-propene:acid potential energy surfaces, we refer to the hydrogen-bonded molecule 1-oxo-3-hydroxy-2-propene as **1** and name the complexes **1**:LiH(OH), **1**:LiH(ts), and **1**:LiH(CO), where **1**:LiH(OH) indicates that the acid LiH interacts with the hydroxyl oxygen, **1**:LiH(CO) indicates that the interaction with the acid occurs at the carbonyl oxygen, and **1**:LiH(ts) identifies the transition structure. These complexes are illustrated in Scheme 1.

### 2.1. Ground State Structures and Binding Energies 

Table S1 of the Supporting Information provides the structures, total energies, and molecular graphs of the complexes of 1-oxo-3-hydroxy-2-propene with the Lewis acids LiH, LiF, BeH_2_, and BeF_2_. The binding energies, selected distances, and the H–O–O angles in these complexes are reported in Table 1. For the equilibrium complexes, the binding energies range from 65 kJ·mol^−1^ for the complex **1**:LiH(OH) to 100 kJ·mol^−1^ for **1**:BeF_2_(CO). For each acid, the binding energies decrease in the following order:

**1**:acid(CO) > **1**:acid (OH) > **1**:acid(ts).

When the interaction with the acid occurs at the carbonyl oxygen, the order of decreasing binding energy with respect to the acid is:

BeF_2_ > BeH_2_ ≈ LiF > LiH 

However, when the interaction occurs at the hydroxyl oxygen, the order is: 

BeF_2_ > LiF > BeH_2_ > LiH.

**Figure 1:**
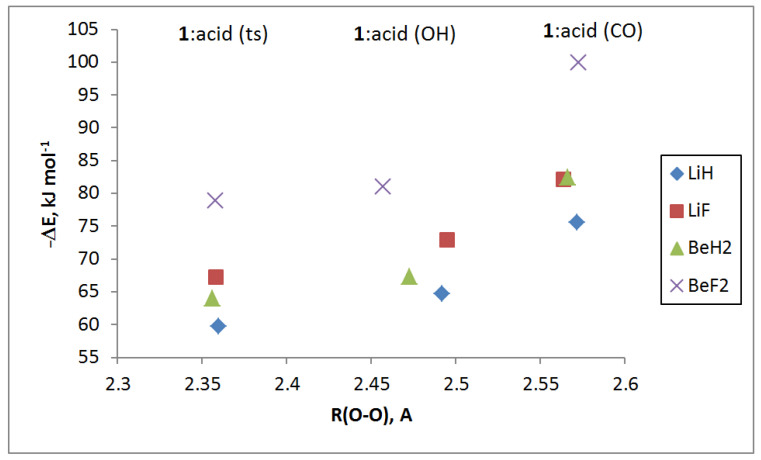
Binding energies versus the O–O distance for **1**:acid (OH), **1**:acid (ts), and **1**:acid (CO) complexes as a function of the acid.

The differences among the binding energies of the equilibrium complexes with the acid at C=O versus O–H range from 9 kJ·mol^−1^ for the complexes with LiF as the acid to 19 kJ·mol^−1^ when BeF_2_ is the acid. Figure 1 provides a representation of the binding energies versus the O–O distance for these complexes and transition structures as a function of the acid. It is interesting to note that the binding energies of the transition structures are very similar to those of the complexes with the acid at the O–H group. Moreover, the binding energies of **1**:LiF(CO) and **1**:BeH_2_(CO) differ by only 0.5 kJ·mol^−1^.

**Figure 2:**
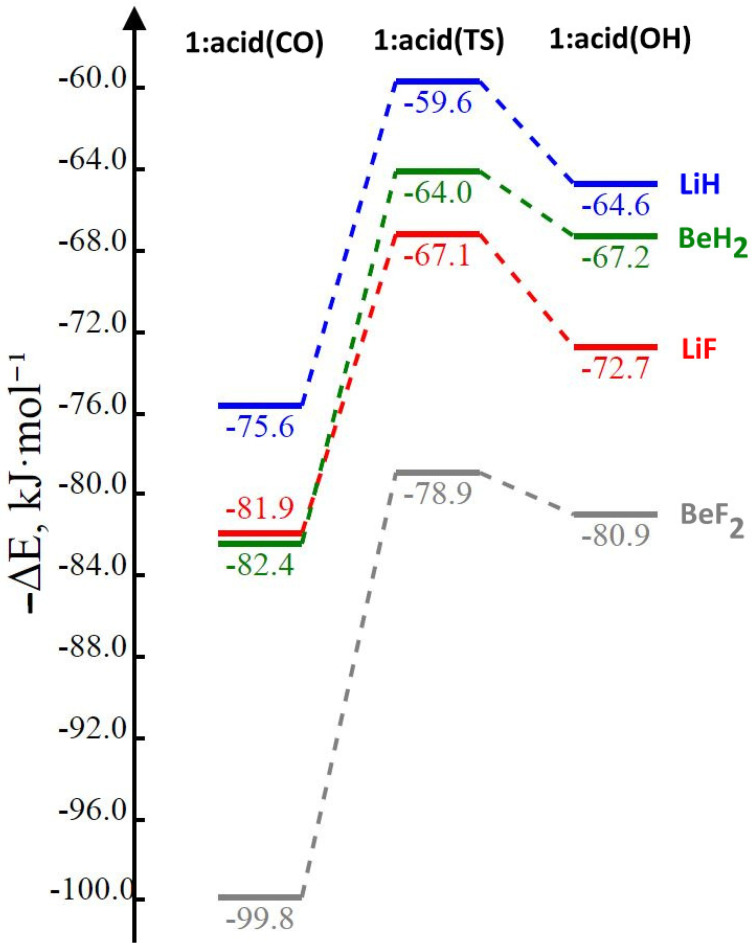
Binding energies of equilibrium and transition structures as a function of the nature of the complex. From these data, the barriers to interconverting the two equilibrium complexes can be readily obtained.

There are many approaches to representing the binding energies of a series of complexes. One of the most interesting and informative can be found in Figure 2, which provides a diagram illustrating the binding energies and the relative binding energies of complexes and transition structures **1**:acid(CO), **1**:acid(ts), and **1**:acid(OH). The transition structures present the barriers that separate the equilibrium structures with the acid at C=O from the structures with the acid at O–H. This barrier is 12 kJ·mol^−1^ for the isolated parent molecule **1**. Interaction of the acid with the C=O group increases the barrier to between 15 and 21 kJ·mol^−1^, while interaction at the O–H group decreases the barrier to between 2 and 6 kJ·mol^−1^. These latter barriers and the energy differences indicate that the population of the isomer with the acid at the carbonyl group would be the greater than 98% at room temperature.

**Figure 3:**
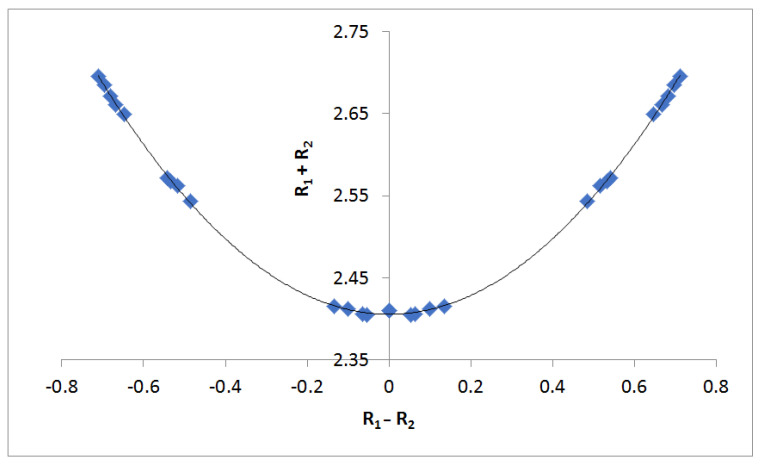
(R_1_ + R_2_) vs. (R_1_ − R_2_) from the Steiner–Limbach relationship.

The O–O distances across the hydrogen bond in the complexes **1**:acid with hydrogen bond formation at the C=O group increase slightly relative to isolated **1**, which has an O–O distance of 2.56 Å. However, when hydrogen bond formation occurs at the O–H group, the O–O distance decreases to between 2.46 to 2.50 Å. As expected, the shortest O–O distances are found in the transition structures for proton transfer, where they decrease to 2.36 Å. An excellent second-order relationship can be obtained when the sum of the O–H distances (R_1_ + R_2_) in each system is compared to the difference (R_1_ − R_2_) using the Steiner–Limbach relationship [45,46]. The points with the largest (R_1_ + R_2_) values in Figure 3 correspond to the **1**:acid(OH) complexes, the intermediate ones to the **1**:acid(CO) complexes, and the shortest to the **1**:acid(TS) complexes. This figure illustrates that the hydrogen-bonded H atom tends to be centered between the two oxygen atoms as they approach each other. The correlation coefficient of the second-order trending in Figure 3 is 0.9996.

The hydrogen bonds in all complexes are nonlinear. The deviation from linearity is 20° in isolated **1** and ranges from 17° to 22° in the complexes. The hydrogen bond approaches closer to linearity in the transition structures, where the deviation decreases to between 11° and 13°.

### 2.2. Orbital Description of the O–H^…^O Hydrogen Bond

**Figure 4:**
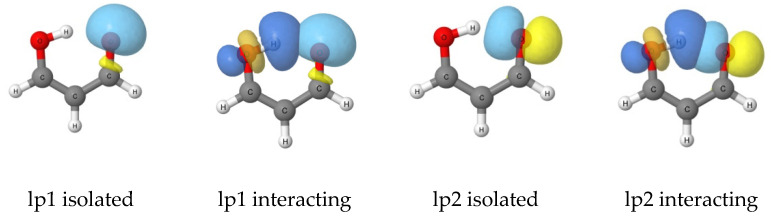
Representations of the lone pair (lp) orbitals of **1** isolated and interacting with the O–H group based on the NBO analysis.

There are two canonical lone pair (lp) orbitals associated with the carbonyl oxygen, both in the isolated base (**1**) and in the **1**:acid complexes, and these are illustrated in Figure 4. The orbital lp1 isolated is a lone-pair orbital on O, which has local σ-type symmetry relative to the C=O bond, extending from the carbonyl oxygen in a direction corresponding to a continuation of the O–H bond. Interaction of the O–H group with this orbital leads to a side-wise overlap of a *p*-type orbital on the O–H group with the C=O lp1 orbital. The orbital lp2 is a local π-type orbital on **1,** which is perpendicular to the C=O bond and directed toward the O–H group of **1** with which it interacts. This orbital extends on both sides of the C=O group, where it may also interact with an acid through the lobe of the *p*-type orbital which extends in this direction. This observation is consistent with the greater binding energies of complexes with the base interacting with **1** at the C=O group compared to those with the base interacting at the O–H group.

### 2.3. Charge-Transfer Energies

**Figure 5:**
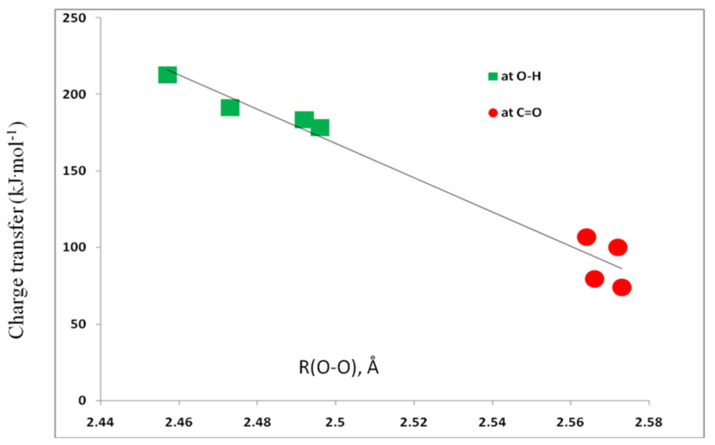
Charge-transfer energies versus the O–O distance.

The complexes **1**:acid are stabilized by charge-transfer interactions. The nature of charge transfer and the associated charge-transfer energies are reported in Table 2. Given the nature of the lone-pair orbitals illustrated in Figure 4, it is not surprising that charge transfer from lp2 is the dominant charge-transfer interaction, with energies ranging from 158 kJ·mol^−1^ in **1**:LiF(OH) to 189 kJ·mol^−1^ **1**:BeF_2_(OH). The charge-transfer energies involving lp1 are much less, with values between 15 and 31 kJ·mol^−1^. The total charge-transfer energies vary from 100 to 213 kJ·mol^−1^. It is interesting to note that the strongest complexes occur in **1**:acid(CO) (Table 1 and Figure 2), while the strongest intramolecular hydrogen bond can be seen in **1**:acid(OH). Figure 5 illustrates a linear dependence of these energies on the O–O distance, with a correlation coefficient of 0.965.

### 2.4. Electron Density Analyses

The electron densities of the equilibrium and transition structures were analyzed using the quantum theory of atoms in molecules (QTAIM) methodology. All complexes have two bond critical points. In the equilibrium structures, the first bond critical point is associated with the covalent O–H bond with ρ_BCP_ values of 0.30 au, while the second refers to the hydrogen bond with ρ_BCP_ values around 0.05 au. The transition structures have intermediate values of ρ_BCP_ between 0.15 to 0.20 au. The ▽^2^ρ_BCP_ values are negative for the covalent O–H bonds in the equilibrium and transition structures but positive for the hydrogen bonds. The total energy densities, H_BCP,_ are negative in all structures. Thus, all O–H contacts have some covalent character. The covalency is of medium strength in the equilibrium complexes that have a positive ▽^2^ρ_BCP_ and a negative value of H_BCP_, while the transition structures have much stronger covalent interactions with a negative value of both ▽^2^ρ_BCP_ and H_BCP_ [47,48]. Excellent exponential correlations are obtained between ρ_BCP_ and H_BCP_ versus the interatomic distance, as illustrated in Figure S1, in agreement with other reports of these parameters as descriptors of intermolecular interactions [49,50,51].

### 2.5. Spin–Spin Coupling Constants 

The total spin–spin coupling constants ^2h^J(O–O), ^1h^J(H–O), and ^1^J(O–H) are given in Table 3, and the paramagnetic spin–orbit (PSO), diamagnetic spin–orbit (DSO), Fermi contact (FC), and spin–dipole (SD) components are reported in Table S2 of the Supporting Information. For the equilibrium ground states of the complexes, the PSO, FC, and SD components of ^2h^J(O–O) are positive. Both the PSO and SD components of ^2h^J(O–O) are non-negligible, with the PSO component having values comparable to those of the FC term when interaction with the acid occurs at the carbonyl group. Even though the FC terms for the transition structures have values that are greater and closer to total J, the FC terms are poor approximations to the total coupling constant ^2h^J(O–O).

Coupling constants ^1h^J(H–O) are also small and positive for the equilibrium structures and are dominated by the FC terms. The PSO terms are smaller and positive for the equilibrium structures, but these are partially canceled by the negative DSO and SD terms. The net result is that the FC terms differ from ^1h^J(H–O) by about 1 Hz. The coupling constant ^1^J(O–H) is negative and has a significantly greater absolute value than ^2h^J(O–O) and ^1h^J(H–O) since it refers to a covalent O–H bond. Values of this coupling constant are dominated by the negative FC terms, while the PSO terms make smaller, non-negligible negative contributions to ^1^J(O–H) in the equilibrium structures but positive contributions in the transition structures. Thus, all terms should be included in determining ^1^J(O–H).

#### 2.5.1. ^2h^J(O–O)

**Figure 6:**
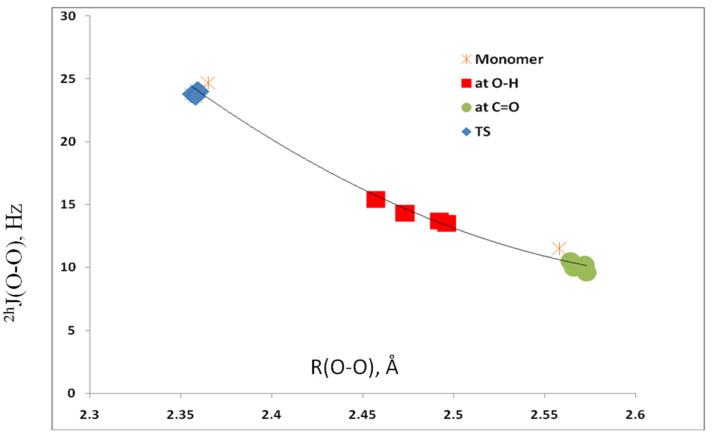
^2h^J(O–O) versus the O–O distance for complexes **1**:acid.

Spin–spin coupling constants ^2h^J(O–O) across the O–H^…^O hydrogen bond are reported in Table 3. This coupling constant has a value of 12 Hz in the isolated monomer **1** and then decreases to about 10 Hz in the complexes when the acid interacts with **1** at the C=O bond. When interaction occurs at the O–H bond, ^2h^J(O–O) increases to between 13 and 15 Hz. The value of ^2h^J(O–O) obviously depends on the O–O distance, so it is not surprising that ^2h^J(O–O) increases to about 24 Hz in the transition structures that have the shortest O–O distances. These relationships are seen most easily in Figure 6, which is a plot of ^2h^J(O–O) versus the O–O distance. The second-order trendline has a correlation coefficient of 0.992.

#### 2.5.2. ^1h^J(H–O) 

**Figure 7:**
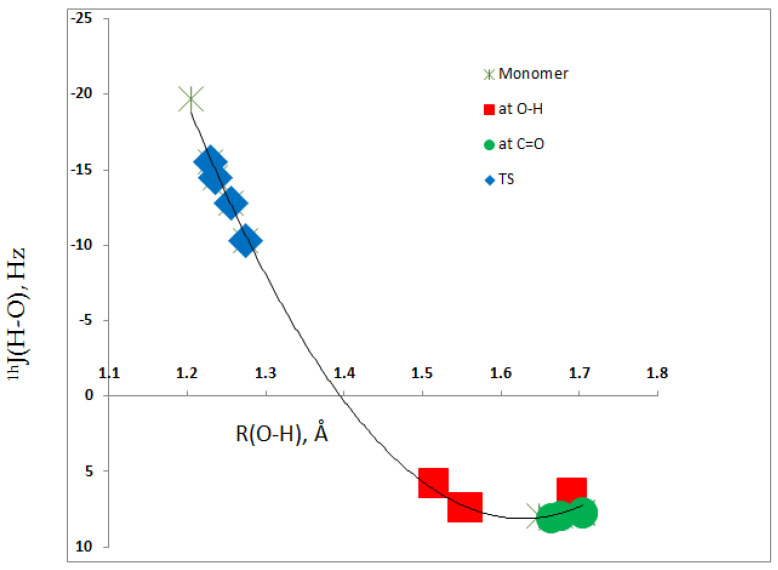
^1h^J(O–H) versus the O–H distance for complexes **1**:Acid.

The values of the second coupling constant ^1h^J(H–O) across the hydrogen bond are also reported in Table 3. Its value of 7.9 Hz in **1** changes minimally upon complex formation, ranging from 7.7 to 8.1 Hz when complexation occurs at the O–H group. It decreases to between 5.8 and 7.4 Hz when the acid interacts at the C=O group. Much larger changes are observed in the transition structures as the H–O distance across the hydrogen bond contracts, and ^1h^J(H–O) has a value of −19.7 Hz in **1**. This coupling constant varies from −10.3 Hz in the complex with BeF_2_ as the acid to −15.5 Hz with LiF as the acid. The dependence of ^1h^J(H–O) on the H–O distance is illustrated in Figure 7, which has a second-order trendline with a correlation coefficient of 0.997. The vertical ^1h^J(H–O) axis was reversed to account for the negative magnetogyric ratio of ^17^O. This plot also illustrates the changing nature of the hydrogen bond, from a traditional hydrogen bond in the equilibrium complexes with bond formation at the O–H or C=O groups to a proton-shared hydrogen bond in the transition structures. 

#### 2.5.3. ^1^J(O–H)

**Figure 8:**
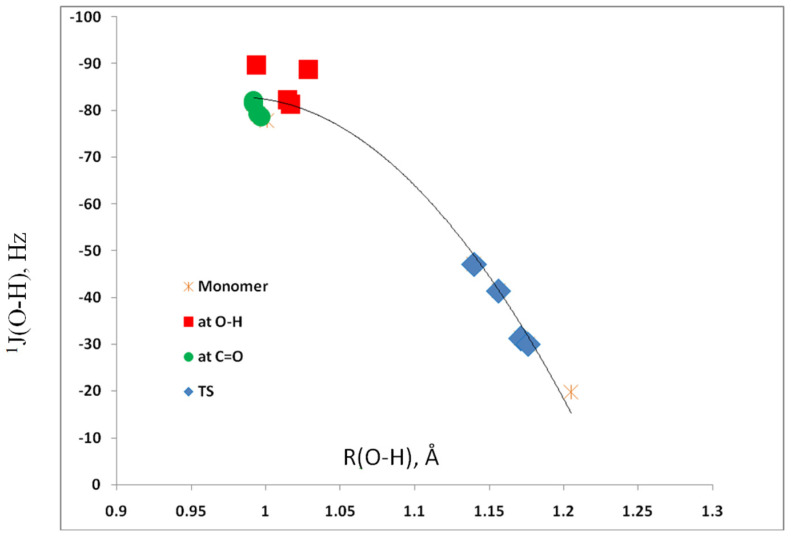
^1^J(O–H) versus the O–H distance for complexes **1**:acid.

The third coupling constant associated with the O–H^…^O hydrogen bond is ^1^J(O–H) for the proton donor O–H group. Its value in **1** of −78 Hz minimally changes in complexes in which the acid interacts with the C=O group, varying between −79 and −82 Hz. However, interaction with the O–H group leads to a significant increase in the absolute value of ^1^J(O–H) to between −81 and −90 Hz. As the O–H distance increases dramatically in the transition structures, ^1^J(O–H) decreases significantly in absolute value to −20 Hz in **1** and from −30 to −47 Hz in the complexes with the acids. Once again, the variation in this coupling constant can be readily seen in the plot of ^1^J(O–H) versus the O–H distance, shown in Figure 8. The correlation coefficient of the second-order trendline is 0.973.

## 3. Methods

The structures of the isolated monomer 1-oxo-3-hydroxy-2-propene; the acids LiH, LiF, BeH_2_, and BeF_2_; and the complexes of 1-oxo-3-hydroxy-2-propene with the acids were optimized at second-order Møller–Plesset perturbation theory (MP2) [52,53,54,55] with the aug’-cc-pVTZ basis set [56]. This basis set was derived from the Dunning aug-cc-pVTZ basis set [57,58] by removing diffuse functions from H atoms. Searches were made of the 1-oxo-3-hydroxy-2-propene:acid potential surfaces for equilibrium structures and transition structures. Frequencies were computed to confirm that the optimized structures are indeed equilibrium structures with no imaginary frequencies and that the transition structures have one imaginary frequency along the path that connects two equilibrium structures. Optimization and frequency calculations were performed using the Gaussian 16 program [59]. The binding energies of the equilibrium complexes were computed as −ΔE for the reaction that forms these complexes from the isolated monomers. 

The natural bond orbital (NBO) method [60] was used to obtain the stabilizing charge-transfer interactions using the NBO-6 program [61]. Since MP2 orbitals are nonexistent, the charge-transfer interactions were computed using the B3LYP functional with the aug’-cc-pVTZ basis set at the MP2/aug’-cc-pVTZ geometries so that at least some electron correlation effects could be included. The atoms in molecules (AIM) methodology [62,63,64,65] was used to produce the molecular graphs of the complexes, employing the AIMAll program [66]. The molecular graph identifies the location of electron density features of interest, including the electron density (ρ) maxima associated with the various nuclei and saddle points that correspond to bond critical points (BCPs). The zero gradient line that connects a BCP with two nuclei is the bond path.

Spin–spin coupling constants were evaluated using the equation-of-motion coupled cluster singles and doubles (EOM-CCSD) method in the CI (configuration interaction)-like approximation [67,68] with all electrons correlated. For these calculations, the Ahlrichs [69] qzp basis set was placed on ^13^C, ^17^O, and ^19^F atoms, the hybrid basis set developed previously on ^7^Li and ^9^Be [70], and the qz2p basis set on the hydrogen-bonded ^1^H atom. The Dunning cc-pVDZ basis was placed on the remaining ^1^H atoms. All terms that contribute to the total coupling constant, namely, the paramagnetic spin–orbit (PSO), diamagnetic spin–orbit (DSO), Fermi contact (FC), and spin–dipole (SD) were evaluated. The EOM-CCSD calculations were performed using ACES II [71] on the HPC cluster Owens at the Ohio Supercomputer Center.

## 4. Conclusions

Ab initio MP2/aug’-cc-pVTZ calculations were carried out to identify and characterize hydrogen-bonded equilibrium structures and transition structures on the 1-oxo-3-hydroxy-2-propene:acid (**1**:acid) potential energy surfaces, with the acids LiH, LiF, BeH_2_, and BeF_2_. The results of these calculations support the following statements:

1. Two equilibrium structures, one with the acid interacting with the C=O group and the other with the interaction occurring at the O–H group, exist on all surfaces. These structures are separated by transition structures that present the barriers to the interconversion of the two equilibrium structures.
2. The binding energies of these complexes vary between 65 and 100 kJ·mol^−1^, with binding at the C=O group preferred by 10 to 20 kJ·mol^−1^.
3. The barrier to interconverting the equilibrium structures with the acid at the C=O group to the structure with the acid at the O–H group is 12 kJ·mol^−1^ in isolated **1** and increases to between 15 and 21 kJ·mol^−1^ in the complexes. The reverse barriers range from 2 to 6 kJ·mol^−1^. Thus, only structures with the acid interacting at the C=O group would be experimentally observed, even at low temperatures.
4. Charge-transfer stabilizes the **1**:acid complexes. The greater charge-transfer interactions involve electron donation from an oxygen lone pair orbital on the C=O group to an antibonding pi-type orbital on the O–H group.
5. EOM-CCSD spin–spin coupling constants ^2h^J(O–O), ^1h^J(H–O), and ^1^J(O–H) were computed for all equilibrium and transition structures. Plots of ^2h^J(O–O) versus the O–O distance, ^1h^J(H–O) versus the H–O distance across the hydrogen bond, and ^1^J(O–H) versus the covalent O–H bond distance of **1** exhibit a second-order dependence of the coupling constant on the corresponding distance, with very high correlation coefficients.

## Figures and Tables

**Table 1:** Binding energies (−ΔE, kJ·mol^−1^), distances R (Å), and H–O–O angles (<, ^o^) for complexes of C_3_H_4_O_2_ with acids.

Complex	−ΔE	R(O···O)	R(O–H)	R(O···H)	<H–O^…^O
Isolated monomer C_3_H_4_O_2_	0.0	2.558	1.001	1.648	19.5
ts isolated ^a^	−11.6 ^a^	2.365	1.205	1.205	11.1
C_3_H_4_O_2_:LiH at C=O	75.6	2.572	0.995	1.677	20.6
at O–H	64.6	2.492	1.017	1.551	17.3
ts	59.6	2.360	1.171	1.235	11.5
C_3_H_4_O_2_:LiF at C=O	81.9	2.564	0.997	1.664	20.2
at O–H	72.7	2.496	1.015	1.557	17.4
ts	67.1	2.359	1.176	1.229	11.4
C_3_H_4_O_2_:BeH_2_ at C=O	82.4	2.566	0.992	1.704	23.0
at O–H	67.2	2.473	0.994	1.691	22.5
ts	64.0	2.356	1.156	1.256	12.9
C_3_H_4_O_2_:BeF_2_ at C=O	99.8	2.573	0.992	1.704	23.0
at O–H	80.9	2.457	1.029	1.514	18.2
ts	78.9	2.358	1.140	1.275	13.3

^a^ The transition structure is 11.6 kJ·mol^−1^ less stable than the equilibrium C_3_H_4_O_2_ structure.

**Table 2:** Intramolecular charge-transfer stabilization energies (kJ·mol^−1^) for complexes of C_3_H_4_O_2_ with acids.

Complex	O_lp1_→σ*O–H	O_lp2_→σ*O–H	Total
Isolated monomer C_3_H_4_O_2_	16.9	105.5	122.5
C_3_H_4_O_2_:LiH at C=O	15.2	84.9	100.1
at O–H	21.0	162.5	183.6
C_3_H_4_O_2_:LiF at C=O	15.1	91.7	106.8
at O–H	20.7	157.7	178.4
C_3_H_4_O_2_:BeH_2_ at C=O	26.1	53.6	79.7
at O–H	22.9	168.6	191.5
C_3_H_4_O_2_:BeF_2_ at C=O	30.5	43.6	74.1
at O–H	24.1	188.7	212.8

**Table 3:** Spin–spin coupling constants ^2h^J(O–O), ^1h^J(H–O), and ^1^J(O–H) (Hz) for the O–H**^…^**O hydrogen bond in complexes C_3_H_4_O_2_:Acid.

Complex	^2h^J(O–O)	^1h^J(H–O)	^1^J(O–H)
Isolated monomer C_3_H_4_O_2_	11.5	7.9	−77.7
Isolated ts	24.7	−19.7	−19.7
C_3_H_4_O_2_:LiH at C=O	10.2	7.9	−79.2
at O–H	13.7	7.4	−81.3
ts	24.0	−14.5	−31.2
C_3_H_4_O_2_:LiF at C=O	10.5	8.1	−78.6
at O–H	13.5	7.4	−82.2
ts	24.1	−15.5	−30.0
C_3_H_4_O_2_:BeH_2_ at C=O	10.0	7.8	−81.4
at O–H	14.3	6.4	−89.7
ts	23.8	−12.8	−41.4
C_3_H_4_O_2_:BeF_2_ at C=O	9.6	7.7	−82.1
at O–H	15.4	5.8	−88.7
ts	23.6	−10.3	−47.1

## Data Availability

The data presented in this study are available in the article and in the Supplementary Material.

## Supplementary Materials

The following are available online, Table S1: Structures (Å), total energies (au), and molecular graphs of 1-oxo-3-hydroxy-2-propene:acid complexes; Figure S1: Relationship between electron densities at the O–H hydrogen bonds and interatomic distances; Table S2: Components of spin–spin coupling constants ^2h^J(O–O), ^1h^J(H–O), and ^1^J(O–H) (Hz).
