# Supplementary material for: Perturbing the O–H…O Hydrogen Bond in 1-oxo-3-hydroxy-2-propene
Source: Molecules. 2021 May 21;26(11):3086. doi: 10.3390/molecules26113086 (PMC8196739; doi:10.3390/molecules26113086)
Supplement: Supplementary file 1 [file molecules-26-03086-s001.zip › molecules-1193215-supplementary.pdf]

Supporting Information for

# Perturbing the O-H···O hydrogen bond in 1-oxo-3-hydroxy-2-propene

Ibon Alkorta,*[a] José Elguero,[a] and Janet E. Del Bene,*[b]

[a] *Instituto de Química Médica (IQM-CSIC), Juan de la Cierva, 3, E-28006 Madrid, Spain*

[b] *Department of Chemistry, Youngstown State University, Youngstown, Ohio 44555, USA*

Pgs. S2 – S6: Table S1. Structures (Å), total energies (au), and molecular graphs of 1-oxo-3-hydroxy-2-propene:Acid complexes

Pg. S7 Fig. S1. Relationship between electron densities at the O-H hydrogen bonds and interatomic distances.

Pg. S8 – S10: Table S2. Components of spin-spin coupling constants $^{2h}J$(O-O), $^{1h}J$(H-O), and $^{1}J$(O-H) (Hz)

Table S1. Structures (Å), total energies (au), and molecular graphs of 1-oxo-3-hydroxy-2-propene:Acid complexes

| | |
|---|---|
| 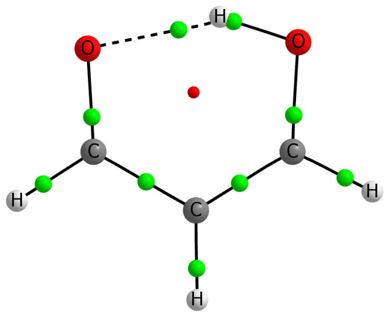 | **1**<br>MP2= -266.70194280 NIMAG= 0<br>C,-0.0985920137,0.,0.0464953349<br>O,-0.0387775045,0.,1.2894512904<br>H,0.836805299,0.,-0.5285506099<br>C,-1.3355459477,0.,-0.6807835651<br>H,-1.3401544589,0.,-1.7589277237<br>C,-2.5131732456,0.,0.0049034509<br>H,-3.4766886818,0.,-0.4906817834<br>O,-2.5964771066,0.,1.3233776836<br>H,-1.6483545336,0.,1.6447159223 |
| 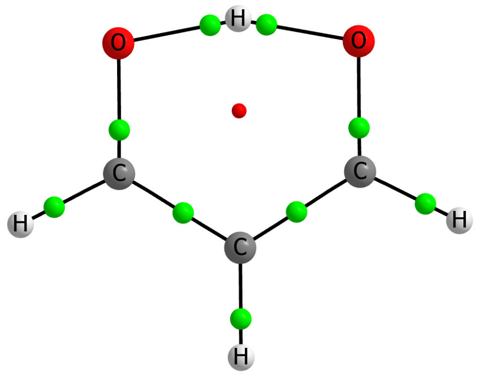 | **1**(ts)<br>MP2= -266.69753851 NIMAG= 1<br>C,1.1854240801,0.,-0.3727246333<br>O,1.1825128883,0.,0.9081519642<br>H,2.1590853689,0.,-0.8618542603<br>C,0.,0.,-1.1089202091<br>H,0.,0.,-2.1855588128<br>C,-1.1854240801,0.,-0.3727246333<br>H,-2.1590853689,0.,-0.8618542603<br>O,-1.1825128883,0.,0.9081519642<br>H,0.,0.,1.1401074708 |
| 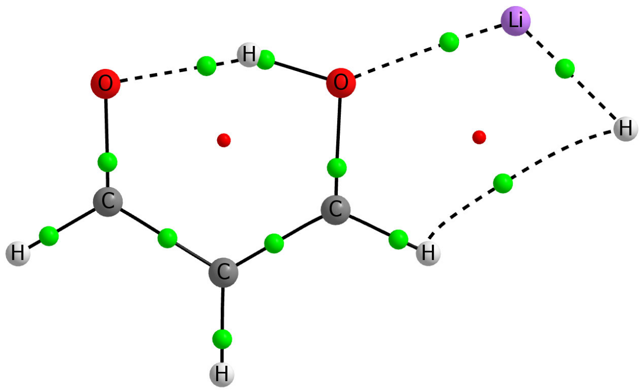 | **1**:LiH(OH)<br>MP2= -274.74064245 NIMAG= 0<br>C,-0.1625693391,0.,-0.0365729891<br>O,0.0603365221,0.,1.1897078029<br>H,0.6894810041,0.,-0.7256180356<br>C,-1.4895771608,0.,-0.5867000297<br>H,-1.6449084565,0.,-1.6531536029<br>C,-2.5577080754,0.,0.2508799595<br>H,-3.5991239563,0.,-0.0510538323<br>O,-2.4002201358,0.,1.5851991902<br>H,-1.3934330233,0.,1.7295174921<br>Li,-4.1218204159,0.,2.5180736493<br>H,-5.4553335727,0.,1.5711424971 |

| | |
|---|---|
| 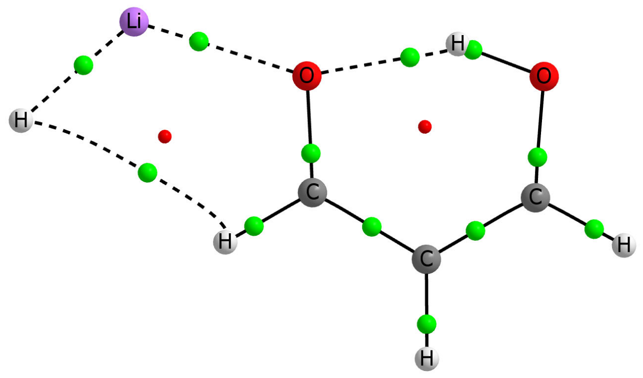 | **1**:LiH(CO)<br>MP2= -274.74482785 NIMAG= 0<br>C,-2.4687496639,0.,-0.0769525076<br>O,-2.7758111196,0.,1.2036433539<br>H,-3.3345132803,0.,-0.7279551233<br>C,-1.1907052801,0.,-0.5601314554<br>H,-1.0213938657,0.,-1.6242316934<br>C,-0.0867566574,0.,0.335157743<br>H,0.9351987577,0.,-0.055904677<br>O,-0.2332587632,0.,1.5888012568<br>H,-1.907140805,0.,1.6886934289<br>Li,1.5251202794,0.,2.4441707718<br>H,2.9125538613,0.,1.5706287515 |
| 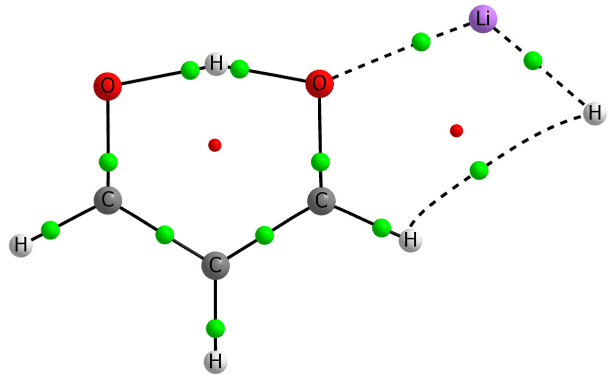 | **1**:LiH(ts)<br>MP2= -274.73873745 NIMAG= 1<br>C,1.1942333168,0.,-0.3666021708<br>O,1.1946046057,0.,0.9051655943<br>H,2.1642978563,0.,-0.8636968214<br>C,-0.002479598,0.,-1.1066491276<br>H,-0.0004511476,0.,-2.183177878<br>C,-1.1830736394,0.,-0.3946669917<br>H,-2.1733544434,0.,-0.8444681028<br>O,-1.165329557,0.,0.9162931086<br>H,-0.0168727554,0.,1.14501648<br>Li,-2.9864156072,0.,1.6254573952<br>H,-4.2296604755,0.,0.5580904478 |
| 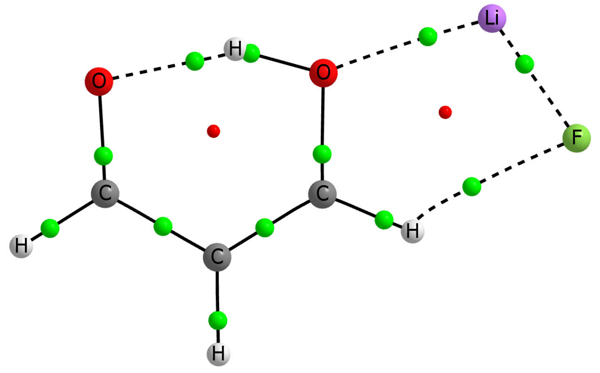 | **1**:LiF(OH)<br>MP2= -373.99985235 NIMAG= 0<br>C,-0.1868943779,0.,-0.0443748221<br>O,0.0735448291,0.,1.1752030266<br>H,0.6450667905,0.,-0.7580829713<br>C,-1.5271970425,0.,-0.5570094623<br>H,-1.7096038424,0.,-1.6192282101<br>C,-2.5761047335,0.,0.3058879433<br>H,-3.6311973507,0.,0.0463485098<br>O,-2.3791346535,0.,1.6369974003<br>H,-1.3711972258,0.,1.755829412<br>Li,-4.1386920595,0.,2.5125169386<br>F,-5.2734669435,0.,1.3373343365 |

| | |
|---|---|
| 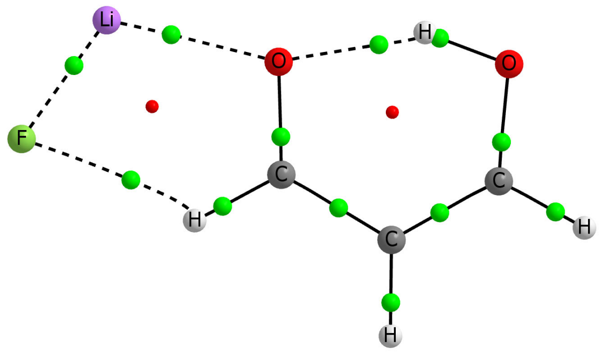 | **1**:LiF(CO)<br>MP2= -374.00336718 NIMAG= 0<br>C,-2.4379447193,0.,-0.086421344<br>O,-2.7860854215,0.,1.1849898015<br>H,-3.2823567667,0.,-0.7650682102<br>C,-1.145141116,0.,-0.5262131884<br>H,-0.9406618196,0.,-1.5841905405<br>C,-0.0692081605,0.,0.4052123676<br>H,0.9724835329,0.,0.065899115<br>O,-0.2657602799,0.,1.654359579<br>H,-1.9294231164,0.,1.6940141589<br>Li,1.5343674033,0.,2.4438535605<br>F,2.7042739268,0.,1.2994845497 |
| 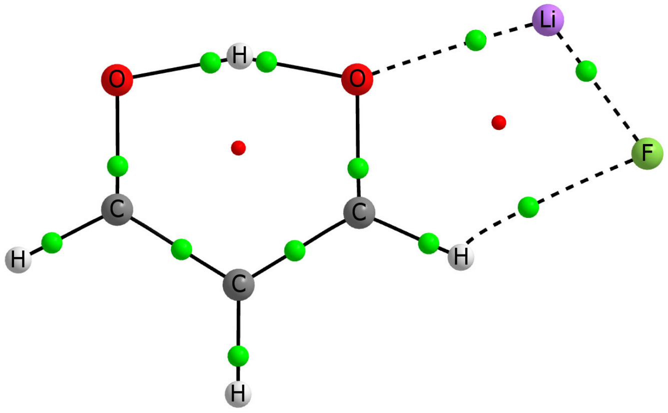 | **1**:LiF(ts)<br>MP2= -373.99770572 NIMAG= 1<br>C,1.1691722505,0.,-0.3807726581<br>O,1.2111784187,0.,0.8924608257<br>H,2.1235751959,0.,-0.9073479227<br>C,-0.0479021539,0.,-1.0815615111<br>H,-0.0781994611,0.,-2.1576965625<br>C,-1.2096366516,0.,-0.3348113787<br>H,-2.221386231,0.,-0.7399798962<br>O,-1.1462181279,0.,0.9753300966<br>H,0.0136257026,0.,1.1678853427<br>Li,-3.0003431412,0.,1.6192118686<br>F,-4.0183672456,0.,0.3380437295 |
| 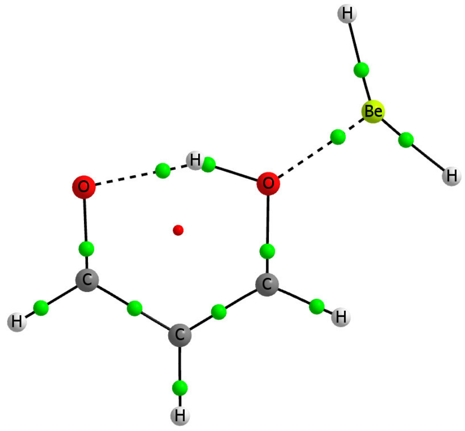 | **1**:$BeH_2$(OH)<br>MP2= -282.56000229 NIMAG= 0<br>C,-0.2368352925,0.,0.0823796046<br>O,-0.1306596403,0.,1.3205502055<br>H,0.6736816788,0.,-0.5262822212<br>C,-1.5152264324,0.,-0.5888868476<br>H,-1.5744655131,0.,-1.6651563818<br>C,-2.6493249233,0.,0.1466683717<br>H,-3.6598308445,0.,-0.2376810847<br>O,-2.5979753075,0.,1.494093531<br>H,-1.6084479128,0.,1.7531549136<br>Be,-3.9523946612,0.,2.5277783459<br>H,-5.0481486291,0.,1.7181318166<br>H,-3.5382421421,0.,3.8154830065 |

| | |
|---|---|
| 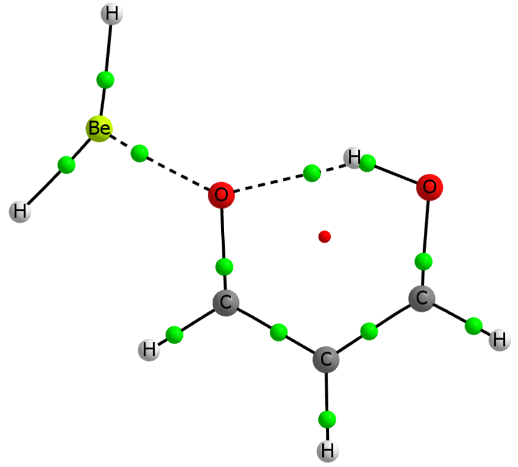  | **1**:$BeH_2$(CO)<br>MP2= -282.56577741 NIMAG= 0<br>C,-2.3862360611,0.,0.0253836566<br>O,-2.6079514417,0.,1.3152815268<br>H,-3.294595433,0.,-0.5652880518<br>C,-1.1437939643,0.,-0.5583038271<br>H,-1.0616581413,0.,-1.6325364343<br>C,0.0173824554,0.,0.2398408147<br>H,1.0125491335,0.,-0.2075297547<br>O,-0.0489762854,0.,1.5060851274<br>H,-1.7201452793,0.,1.7630386993<br>Be,1.3725906976,0.,2.448169067<br>H,2.4362859663,0.,1.5847137772<br>H,1.0864536634,0.,3.7737768489 |
| 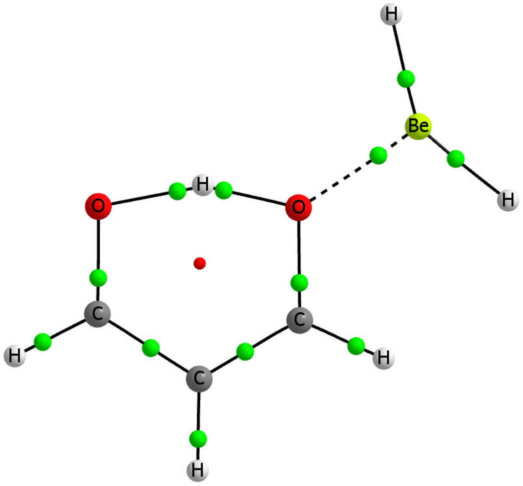  | **1**:$BeH_2$(ts)<br>MP2= -282.55876893 NIMAG= 1<br>C,-0.2688561338,0.,0.0753740415<br>O,-0.1990294664,0.,1.3362231034<br>H,0.6699451274,0.,-0.479102932<br>C,-1.513032767,0.,-0.6075646092<br>H,-1.5631449233,0.,-1.6830154845<br>C,-2.6485886249,0.,0.1544205962<br>H,-3.662372181,0.,-0.2292612647<br>O,-2.5507902196,0.,1.4723844292<br>H,-1.411030119,0.,1.665510547<br>Be,-3.8955090707,0.,2.5121533429<br>H,-5.0055009498,0.,1.7158425002<br>H,-3.493976852,0.,3.80551603 |
| 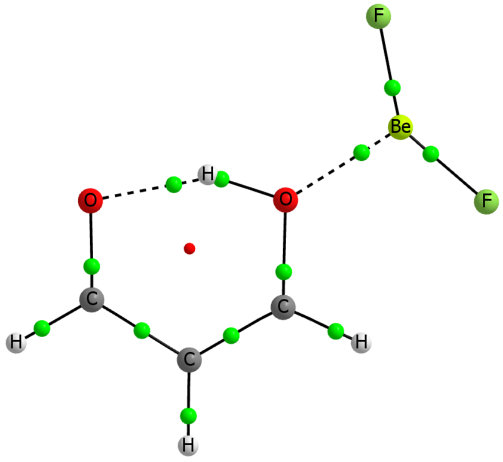  | **1**:$BeF_2$(OH)<br>MP2= -481.07105491 NIMAG= 0<br>C,-0.2344786146,0.,0.0725982921<br>O,-0.1231335874,0.,1.3111971166<br>H,0.6736428066,0.,-0.5387917611<br>C,-1.5161507187,0.,-0.5906946681<br>H,-1.5832708191,0.,-1.6662353524<br>C,-2.643863915,0.,0.1549271674<br>H,-3.6579435217,0.,-0.2195012247<br>O,-2.5729928551,0.,1.501720719<br>H,-1.5736055458,0.,1.7462827031<br>Be,-3.9456611441,0.,2.5183135245<br>F,-5.09574575,0.,1.6637306432<br>F,-3.5646659553,0.,3.8866861005 |

| | |
|---|---|
| 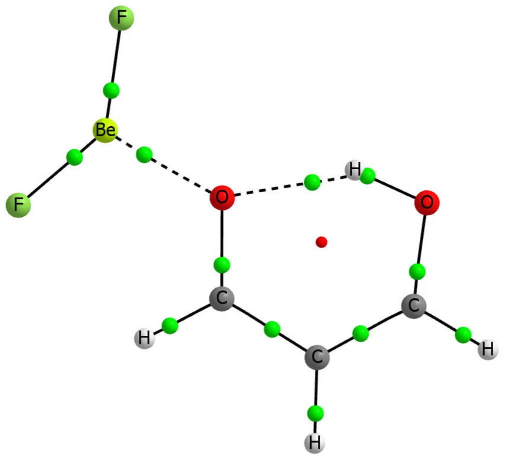 | **1**:$BeF_2$(CO)<br>MP2= -481.07825942 NIMAG= 0<br>C,-2.3917206387,0.,0.0197017568<br>O,-2.6230795281,0.,1.3074790105<br>H,-3.2962101613,0.,-0.5766491269<br>C,-1.146282032,0.,-0.5579458078<br>H,-1.0599004972,0.,-1.6316454851<br>C,0.0121907262,0.,0.2423663568<br>H,1.0065345374,0.,-0.2049221056<br>O,-0.058444529,0.,1.5091477413<br>H,-1.7427937868,0.,1.7655145367<br>Be,1.3660563565,0.,2.4340353569<br>F,2.5046530408,0.,1.5541971788<br>F,1.0909018225,0.,3.8313520377 |
| 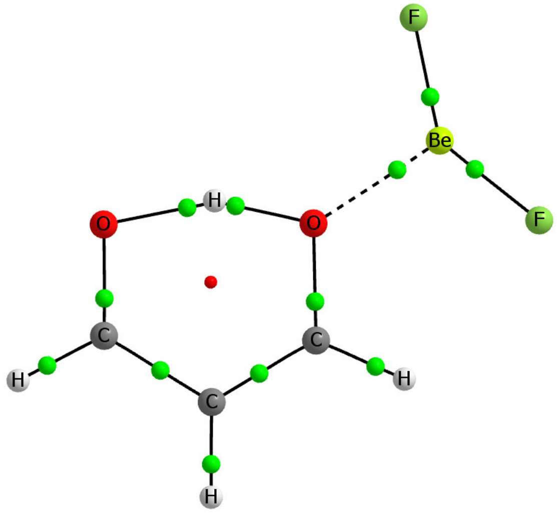 | **1**:$BeF_2$(ts)<br>MP2= -481.07030865 NIMAG= 1<br>C,-0.2593215076,0.,0.0687207484<br>O,-0.1800476272,0.,1.3266141514<br>H,0.6739579374,0.,-0.4956536942<br>C,-1.5115684478,0.,-0.6058663031<br>H,-1.5702595409,0.,-1.6808934405<br>C,-2.6406988778,0.,0.1608476475<br>H,-3.6569712897,0.,-0.2142067287<br>O,-2.5324203971,0.,1.4827776396<br>H,-1.4078406287,0.,1.6708382822<br>Be,-3.8923955935,0.,2.5040665587<br>F,-5.0621495977,0.,1.6715643802<br>F,-3.5170543292,0.,3.8755479187 |

Fig. S1. Relationship between electron densities at the O-H hydrogen bonds and interatomic distances.

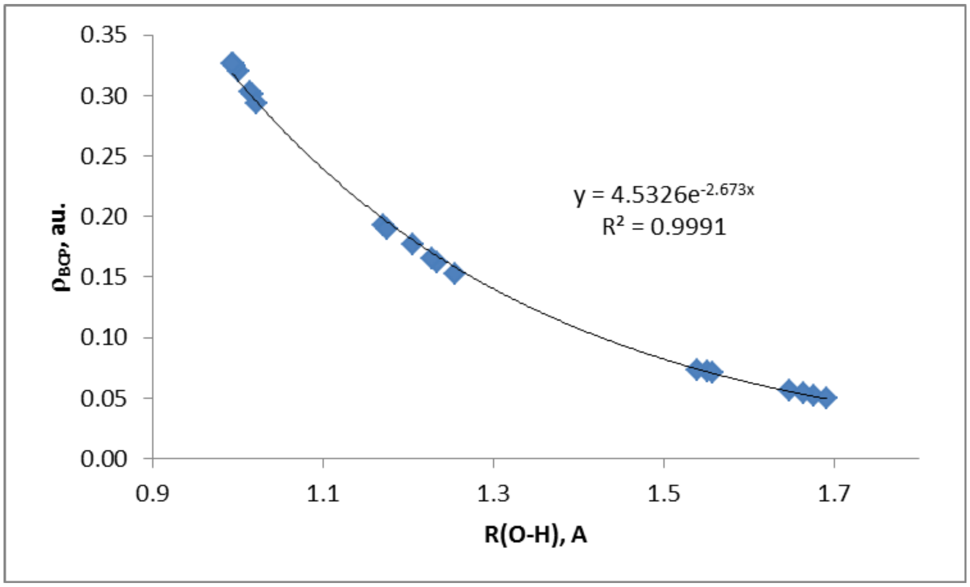


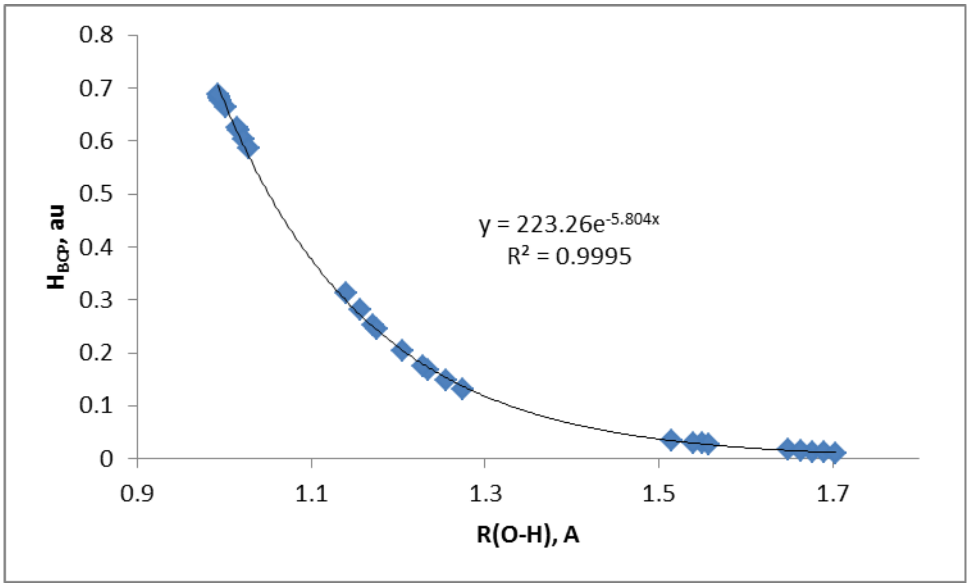

Table S2. Components of spin-spin coupling constants $^{2h}J$(O-O), $^{1h}J$(H-O), and $^{1}J$(O-H) (Hz)

| Monomer/Complex | PSO | DSO | FC | SD | $^{2h}J$(O-O) |
|---|---|---|---|---|---|
| **1** | 5.0 | 0.0 | 4.3 | 2.2 | 11.5 |
| ts | 8.2 | 0.0 | 13.2 | 3.3 | 24.7 |
| | | | | | |
| **1**:LiH at C=O | 4.1 | 0.0 | 4.2 | 1.9 | 10.2 |
| O-H | 4.5 | 0.0 | 7.3 | 1.9 | 13.7 |
| ts | 6.6 | 0.0 | 14.6 | 2.7 | 24.0 |
| | | | | | |
| **1**:LiF at C=O | 4.1 | 0.0 | 4.5 | 1.8 | 10.5 |
| O-H | 4.4 | 0.0 | 7.2 | 1.9 | 13.5 |
| ts | 6.6 | 0.0 | 14.8 | 2.6 | 24.1 |
| | | | | | |
| **1**:$BeH_2$ at C=O | 3.8 | 0.0 | 4.4 | 1.7 | 10.0 |
| O-H | 4.1 | 0.0 | 8.5 | 1.7 | 14.3 |
| ts | 5.8 | 0.0 | 15.6 | 2.3 | 23.8 |
| | | | | | |
| **1**:$BeF_2$ - at C=O | 3.6 | 0.0 | 4.2 | 1.7 | 9.6 |
| O-H | 4.1 | 0.0 | 9.5 | 1.7 | 15.4 |
| ts | 5.6 | 0.0 | 15.8 | 2.3 | 23.6 |

| Monomer/Complex | PSO | DSO | FC | SD | $^{1}J$(O-H) |
|---|---|---|---|---|---|
| **1** | -4.8 | -0.5 | -72.4 | -0.1 | -77.7 |
| ts | 1.3 | -0.7 | -20.0 | -0.2 | -19.6 |
| | | | | | |
| **1**:LiH at C=O | -5.0 | -0.4 | -73.6 | -0.1 | -79.2 |
| O-H | -3.6 | -0.5 | -77.0 | 0.0 | -81.3 |
| ts | 0.8 | -0.7 | -31.1 | -0.1 | -31.2 |
| | | | | | |
| **1**:LiF at C=O | -4.9 | -0.5 | -73.1 | -0.1 | -78.6 |
| O-H | -3.7 | -0.5 | -77.9 | 0.0 | -82.2 |
| ts | 0.9 | -0.7 | -30.0 | -0.1 | -30.0 |
| | | | | | |
| **1**:$BeH_2$ at C=O | -4.8 | -0.4 | -75.9 | -0.2 | -81.4 |
| O-H | -3.0 | -0.6 | -86.1 | 0.0 | -89.7 |
| ts | 0.5 | -0.8 | -41.1 | -0.1 | -41.4 |
| | | | | | |
| **1**:$BeF_2$ at C=O | -4.9 | -0.5 | -76.5 | -0.2 | -82.1 |
| O-H | -2.7 | -0.7 | -85.3 | 0.0 | -88.7 |
| ts | 0.3 | -0.8 | -46.5 | 0.0 | -47.1 |

| Monomer/Complex | PSO | DSO | FC | SD | $^{1h}J$(H-O) |
|---|---|---|---|---|---|
| **1** | 2.2 | -0.7 | 6.6 | -0.2 | 7.9 |
| ts | 1.3 | -0.7 | -20.0 | -0.2 | -19.7 |
| **1** at O | 2.0 | -0.7 | 6.8 | -0.2 | 7.9 |
| at OH | 2.3 | -0.7 | 6.2 | -0.4 | 7.4 |
| ts | 1.6 | -0.7 | -15.0 | -0.3 | -14.5 |
| **1** at O | 2.0 | -0.7 | 6.9 | -0.2 | 8.1 |
| at OH | 2.3 | -0.7 | 6.2 | -0.4 | 7.4 |
| ts | 1.5 | -0.7 | -16.0 | -0.3 | -15.5 |
| **1** at O | 1.8 | -0.7 | 6.8 | -0.2 | 7.8 |
| at OH | 2.2 | -0.7 | 5.3 | -0.4 | 6.4 |
| ts | 1.7 | -0.7 | -13.3 | -0.4 | -12.8 |
| **1** at O | 1.8 | -0.8 | 6.8 | -0.1 | 7.7 |
| at OH | 2.3 | -0.8 | 4.7 | -0.5 | 5.8 |
| ts | 1.9 | -0.8 | -10.9 | -0.4 | -10.3 |
